# Progressive Retinal and Optic Nerve Damage in a Mouse Model of Spontaneous Opticospinal Encephalomyelitis

**DOI:** 10.3389/fimmu.2021.759389

**Published:** 2022-01-24

**Authors:** Laura Petrikowski, Sabrina Reinehr, Steffen Haupeltshofer, Leonie Deppe, Florian Graz, Ingo Kleiter, H. Burkhard Dick, Ralf Gold, Simon Faissner, Stephanie C. Joachim

**Affiliations:** ^1^ Experimental Eye Research Institute, University Eye Hospital, Ruhr-University Bochum, Bochum, Germany; ^2^ Department of Neurology, Ruhr-University Bochum, St. Josef-Hospital, Bochum, Germany

**Keywords:** Neuromyelitis optica, optic nerve, inflammation, demyelination, myelin oligodendrocyte glycoprotein antibodies (MOG-IgG), retinal ganglion cells, microglia, complement system

## Abstract

Neuromyelitis optica spectrum disorder (NMOSD) and myelin oligodendrocyte glycoprotein-antibody-associated disease (MOGAD) are antibody mediated CNS disorders mostly affecting the optic nerve and spinal cord with potential severe impact on the visual pathway. Here, we investigated inflammation and degeneration of the visual system in a spontaneous encephalomyelitis animal model. We used double-transgenic (2D2/Th) mice which develop a spontaneous opticospinal encephalomyelitis (OSE). Retinal morphology and its function were evaluated via spectral domain optical coherence tomography (SD-OCT) and electroretinography (ERG) in 6- and 8-week-old mice. Immunohistochemistry of retina and optic nerve and examination of the retina via RT-qPCR were performed using markers for inflammation, immune cells and the complement pathway. OSE mice showed clinical signs of encephalomyelitis with an incidence of 75% at day 38. A progressive retinal thinning was detected in OSE mice via SD-OCT. An impairment in photoreceptor signal transmission occurred. This was accompanied by cellular infiltration and demyelination of optic nerves. The number of microglia/macrophages was increased in OSE optic nerves and retinas. Analysis of the retina revealed a reduced retinal ganglion cell number and downregulated *Pou4f1* mRNA expression in OSE retinas. RT-qPCR revealed an elevation of microglia markers and the cytokines *Tnfa* and *Tgfb*. We also documented an upregulation of the complement system via the classical pathway. In summary, we describe characteristics of inflammation and degeneration of the visual system in a spontaneous encephalomyelitis model, characterized by coinciding inflammatory and degenerative mechanisms in both retina and optic nerve with involvement of the complement system.

## Background

Chronic inflammatory diseases of the central nervous system (CNS) have profound implications for patients due to the potential development of disability. While Multiple Sclerosis (MS) is the most common cause (1), there is also a spectrum of relatively rare neuroinflammatory and neurodegenerative diseases such as Neuromyelitis optica spectrum disorder (NMOSD) and myelin oligodendrocyte glycoprotein (MOG)-IgG antibody associated disease (MOGAD). 25% of patients with a NMOSD phenotype present with autoantibodies directed against MOG (2). Recently, a robust association of anti-MOG IgG has been found with optic neuritis, myelitis and brainstem encephalitis, as well as with acute disseminated encephalomyelitis (ADEM)-like presentations (3). Hence, MOGAD is now considered as distinct disease entity with differing pathophysiological features compared to NMOSD (3, 4). MOG is located on the outer surface of the oligodendrocytic myelin sheath (5). The target of MOG antibodies are oligodendrocytes; hence the pathogenesis in MOG^+^ patients is different from the astrocytopathy in aquaporin4^+^ (APQ4^+^) NMOSD (6, 7). 

Since loss of vision is perceived as most impairing in daily life (8), further studies regarding the impact of MOG to the visual system, especially the retina, are needed. To better understand the pathogenesis of MOGAD, we took advantage of an animal model of spontaneous opticospinal encephalomyelitis (OSE), characterized by MOG-reactive transgenic T-cells (2D2) and a transgenic B-cell receptor against MOG (Th) (9). OSE mice develop a progressive encephalomyelitis spontaneously, characterized by inflammation and demyelination of the spinal cord and the optic nerve (9, 10). Only few studies investigated retinal damage in NMOSD or MOG models. Zeka et al. observed retinal inflammation in a NMOSD model in rats (11). None of those previous studies focused on the detailed examination of retinal cells in the OSE model, representing MOGAD. Understanding mechanisms of damage in OSE as model of MOGAD is therefore an important step towards identifying potential targets and developing new therapeutic approaches to stop disease progression and avoid loss of vision in affected patients.

In this study, we describe longitudinal dynamics of morphological, functional and structural retinal alterations as well as (immuno-) histochemical changes in the optic nerve in OSE. Data are corroborated by analyses of gene expression alterations of markers of inflammation and complement activation. We show that the retina loses function in accordance with progressive neurodegeneration of retinal ganglion cells (RGCs) in line with inflammation and complement activation, hence informing about potential therapeutic targets in MOGAD.

## Methods

### Animals and Evaluation of Spontaneous Opticospinal Encephalomyelitis

All experiments involving animals were performed in accordance with the ARVO statement for the Use of Animals in Ophthalmic and Vision Research and approved by the animal care committee of North Rhine-Westphalia, Germany (Landesamt für Natur, Umwelt und Verbraucherschutz Nordrhein-Westfalen, Recklinghausen, Germany; file no. 84-02.04.2016.A062). All animals were bred and housed in the animal facility of the Ruhr-University Bochum under environmentally controlled conditions with free access to food and water *ad libitum* in the absence of pathogens.

Both male and female C57BL/6 mice with either MOG-specific T cells (2D2) (12) or MOG-specific B cells (Th) (13) were used for the study. The double-transgenic (2D2/Th) OSE mice resulting from the intercross of the single-transgenic TCR^MOG^ and MOG-specific Ig heavy-chain knock-in (IgH^MOG^) animals spontaneously develop an opticospinal encephalomyelitis with an onset four weeks after birth and an incidence of about 50% (9). The OSE model is a suitable model for MOGAD due to the fact that the demyelinating lesions are restricted to the optic nerve and the spinal cord with histological similarity to human lesions (9). Single-transgenic IgH^MOG^ (Th) mice remain healthy and served as age-matched control animals.

Mice were weighted daily and examined for neurological symptoms using an established 10-point score system: 0=healthy animal, 1=flaccid tail, 2= impaired righting reflex or gait, 3=absent righting, 4=ataxic gait, abnormal position, 5=mild paraparesis, 6=moderate paraparesis, 7=severe paraplegia, 8=tetraparesis, 9=moribund, and 10=death (14).

**Figure 1:**
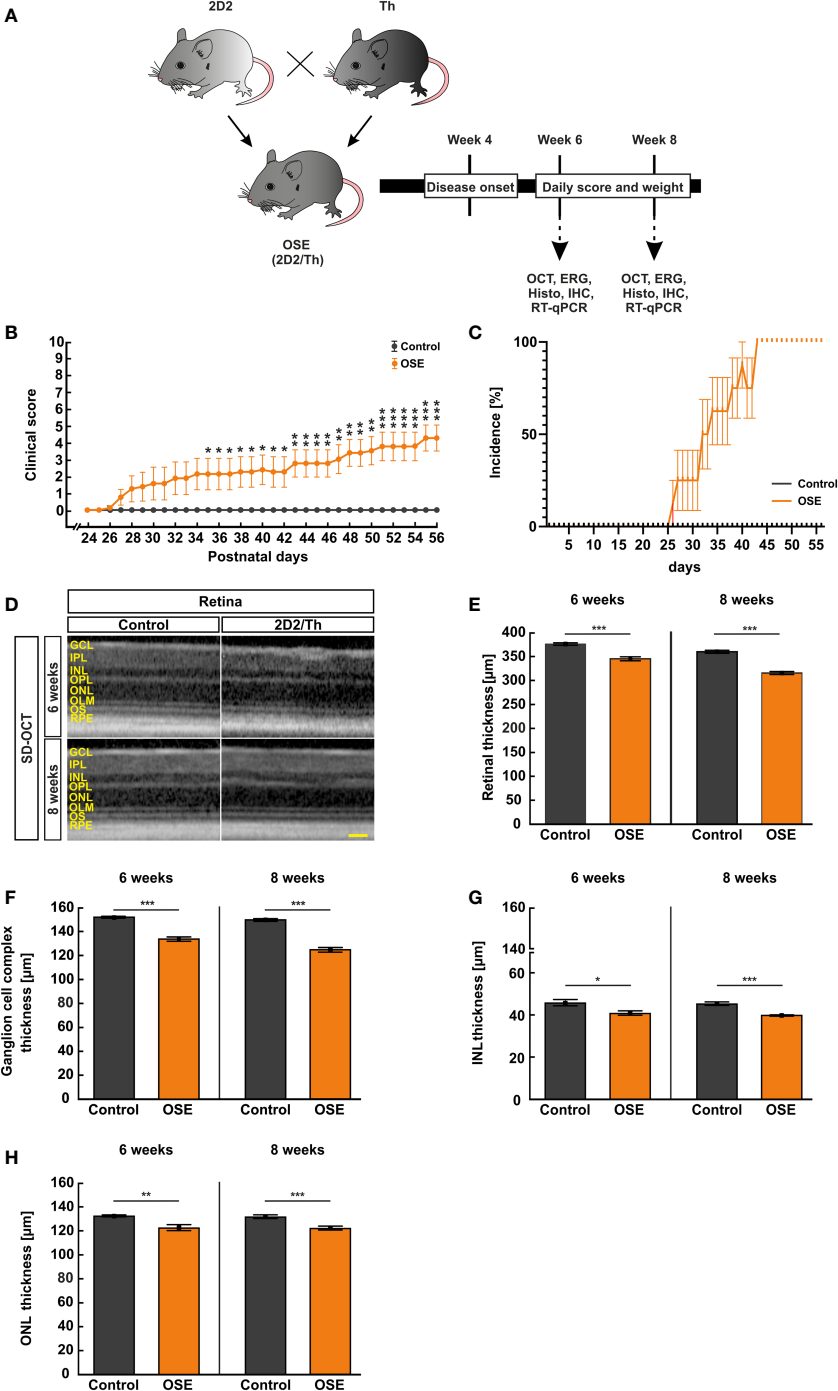
Neurological signs in accordance with structural impairment of the retina. **(A)** Study design. **(B)** OSE mice showed clinical signs of encephalomyelitis with flaccid hind limb paralysis starting at day 26. A significantly higher score was observed in OSE mice while control mice remained healthy. **(C)** Additionally, 50% of OSE mice were affected after 32 days. The incidence increased to 75% at day 38 in OSE mice. **(D)** SD-OCT measurements were performed in six- and eight-week-old mice to evaluate the retinal thickness. **(E)** The morphological analysis of the retina revealed a reduction of the retinal thickness (ganglion cell complex to ONL) in six-week-old OSE animals in comparison to the control group. The reduction of the retinal thickness was also noted after eight weeks. **(F)** The ganglion cell complex thickness (RNFL, GCL and IPL) was reduced by 6.5% in eight-week-old OSE mice. **(G)** The INL thickness decreased by 2.5% between six and eight weeks time point in OSE mice. **(H)** A slight reduction of ONL thickness by 0.4% was seen in OCT analysis in the OSE group. Data are shown as mean ± SEM. ERG, electroretinogram; GCL, ganglion cell layer; Histo, histology; IHC, immunohistochemistry; IPL, inner plexiform layer; INL, inner nuclear layer; OCT, optical coherence tomography; OPL, outer plexiform layer; ONL, outer nuclear layer; OLM, outer limiting membrane; OS, outer segment; RPE, retinal pigment epithelium; RT-qPCR, quantitative real-time polymerase chain reaction. *p < 0.05, **p < 0.01, ***p < 0.001. Scale bar: 200 μm.

At six and eight weeks of age, *in vivo* experiments using SD-optical coherence tomography (SD-OCT) and electroretinography (ERG) measurements were carried out. Afterwards, the eyes and optic nerves were removed for histological and immuno-histochemical analysis or quantitative real-time PCR (RT-qPCR; **Figure 1A**). Subsequent to the preparation, the histological tissues were fixed in 4% paraformaldehyde (Merck, Darmstadt, Germany) for one hour (retina) or two hours (optic nerve), drained in 30% sucrose (VWR, Langenfeld, Germany), embedded in Tissue Tec (Thermo Scientific, Waltham, MA; USA) and frozen at -80°C. While one eye of each animal was used for immunohistological stainings, the other retina was isolated from the surrounding tissue and frozen at -80°C for RT-qPCR.

### *In Vivo* Retinal Imaging Using SD-OCT

We performed SD-OCT measurements in six- and eight-week-old mice (n=8/group) using a Heidelberg Engineering Spectralis OCT device (Heidelberg Engineering, Heidelberg, Germany) which was modified with a +25 dpt lens for murine eyes. The animals were anesthetized with ketamine/xylazine (120/16 mg/kg body weight). Eyes were treated with 5% tropicamide to induce mydriasis before investigation. Additionally, we applied 0.9% NaCl on the eyes before and during the SD-OCT analysis. The mice were placed in front of the SD-OCT. Three horizontal scans and one ring scan were obtained from both eyes of each animal via the Heyex software (Heidelberg Engineering, Heidelberg, Germany) with the following settings: angle 30°, lens 34.5 dpt., art. frames 100, eye size M. The eyes were treated with eye droplets subsequently to the examination to prevent dehydration. The thickness of the retinal layers was measured manually in an axis perpendicular to the individual layers using ImageJ software (National Institute of Health, Bethesda, MD, USA). The middle of the retina as well as two equidistant measurements per side were performed. Hence, five measurements were used to calculate the mean value for each retina and each layer (15–17). The retinal thickness was measured, the total thickness included the retinal nerve fiber layer (RNFL), ganglion cell layer (GCL), inner plexiform layer (IPL), inner nuclear layer (INL), outer plexiform layer, and the outer nuclear layer (ONL). A separate measurement of the ganglion cell complex (RNFL, GCL, and IPL) as well as separate measurements of the INL and ONL thickness were also carried out.

### Electroretinography Recordings

To measure the animals’ retinal function, we used full-field flash ERG (HMsERG system; OcuScience, Henderson, NV, USA) as previously described (18). OSE and control mice, six (OSE: n=5; control: n=6) and eight weeks (n=6/group) of age, were dark adapted the day before the ERGs were performed and narcotized with ketamin/xylazin (120/16 mg/kg body weight) prior to the examination. Eyes were dilated with tropicamide (5%) and topically anesthetized using conjuncain (Bausch&Lomb, Berlin, Germany). A feedback temperature controller (TC-1000; CWE Inc., Ardmore, PA, USA) was used to maintain the body temperature at 37°C. Reference electrodes were placed subcutaneously below both ears and a ground electrode was placed in the base of the tail. Contact lenses with silver thread recording electrodes were attached at the center of the cornea after application of methocel (Omni Vision, Puchheim, Germany). Before measurement, the ERG equipment was covered with a Faraday cage. The scotopic ERG measurements were recorded at 0.1, 0.3, 1, 3, 10, and 25 cd.s/m^2^. Afterwards, the signals were amplificated, digitalized and averaged to evaluate the a- and b-wave using ERGView software (Version 4.380R; OcuScience). Before evaluating the amplitudes of the a- and b-waves, a 50 hz filtering of the data was applied.

### Histopathological Staining and Scoring of the Optic Nerve

We stained longitudinal sections of the optic nerve (4 µm, 3 sections per animal, n=8/group for six and eight weeks) with hematoxylin and eosin (H&E, Merck) or luxol fast blue (LFB; RAL Diagnostics, Martillac Cedex, France). Three images of each cryosection (cranial, medial, caudal) were taken with an Axio Imager M1 microscope (Zeiss, Oberkochen, Germany) at a 400x magnification. The masked pictures were evaluated by two independent examiners regarding cellular infiltration and inflammation using a 4-point scoring system for H&E-stained sections (19): 0=no infiltration, 1=mild infiltration, 2=moderate infiltration, 3=severe infiltration, and 4=massive infiltration with formation of cellular conglomerates. The degree of demyelination in LFB-stained sections was also evaluated by two examiners in 0.5 intervals: 0=no demyelination, 0.5=mild demyelination, 1=moderate demyelination, 1.5=advanced demyelination, and 2=severe demyelination (19).

### Immunohistochemistry of the Optic Nerve and the Retina

Immunohistochemical stainings of longitudinal sections of the optic nerve (4 µm, n=8/group) and of retinal cross-sections (10 µm, n=7-8/group) were performed with appropriate primary and secondary antibodies at six and eight weeks (**Table 1**). We used six sections for each animal. First, sections were blocked for 60 min with a solution containing 10-20% donkey serum with or without 1-10% bovine serum albumin and 0.1-0.3% Triton-X in PBS. Primary antibodies were applied and incubated at room temperature overnight. Then, corresponding secondary antibodies were added for 60 min. Nuclear staining with 4’,6 diamidino-2-phenylindole (DAPI; Serva Electrophoresis, Heidelberg, Germany) was applied to facilitate orientation on the slides. Negative controls were performed for each staining by using the secondary antibodies only.

**Table 1:** Primary and secondary antibodies used for immunohistochemistry on retina and optic nerve sections.

Primary antibodies			Secondary antibodies		
Antibody	Company	Dilution	Antibody	Company	Dilution
Anti-C1q	Abcam	1:200	Donkey anti-rabbit Alexa Fluor 555	Invitrogen	1:500
Anti-C3	Cedarlane	1:500	Donkey anti-rabbit Alexa Fluor 555	Invitrogen	1:500
Anti-GFAP	Millipore	1:2000	Donkey anti-chicken Alexa Fluor 488	Jackson Immuno Research	1:500
Anti-Iba1	Synaptic Systems	1:500	Donkey anti-chicken IgG Cy3	Millipore	1:400
Anti-MAC	Thermo Scientific	1:100	Donkey anti-rabbit Alexa Fluor 555	Invitrogen	1:500
Anti-RBPMS	Millipore	1:500	Donkey anti-rabbit Alexa Fluor 555	Invitrogen	1:500
Anti-Tmem119	Abcam	1:200	Donkey anti-rabbit Alexa Fluor 488	Jackson Immuno Research	1:500

Regarding the optic nerve, three pictures were taken of each longitudinal section (cranial, medial, caudal). Six nerve sections per animal were analyzed (24 photographs/animal). Four pictures were taken of each retinal section (two in the peripheral and two in the central retina) via ApoTome.2 microscope (Zeiss) for all stainings with a 400x magnification. Photos of retinal cross-sections were taken at a distance of 300 and 3100 µm dorsal and ventral to the optic nerve. Six cross-sections per animal were analyzed in total (24 photographs/animal). Afterwards, all images were masked and cut in a predefined size (Corel Paint Shop Pro, V13; Corel Corporation; Ottawa, Canada).

RBPMS^+^, Iba1^+^, Tmem119^+^, C1q^+^, C3^+^, and MAC^+^ (membrane attack complex) cells with a co-localization with DAPI (cell nuclei) were counted using ImageJ software. In the retina, RBPMS^+^ were counted in the GCL. Iba1^+^, Tmem119^+^, C1q^+^, C3^+^, and MAC^+^ cells were quantified in the GCL, IPL, and INL together as well as separately in these three layers.

Regarding GFAP staining, the signal area was measured using an ImageJ macro with the following settings (20): The pictures were transformed into a gray scale (32-bit). After averaging the background subtraction (50 pixel), the lower (14.63) and the upper threshold (252.13) were set. Means per retina were calculated and used for statistical analysis.

### Retinal Quantitative Real‐Time Reverse Transcription Polymerase Chain Reaction

Two retinas from the same group were pooled for RNA preparation (n=5/group for six weeks, n=5-6/group for eight weeks after pooling) and cDNA synthesis was carried out as previously described (21). The designed oligonucleotides for RT-qPCR are shown in **Table 2**. The RT-qPCR was carried out using DyNAmo Flash SYBR Green (Thermo Scientific) on the PikoReal RT-qPCR Cycler (Thermo Scientific). Primer efficiencies of each primer set were calculated based on a dilution series of 5 to 125 ng cDNA. Ct values of the house-keeping genes *β-actin* (*Actb*) and *Cyclophilin* (*Ppid*) were applied for normalization and relative quantification of gene expressions.

**Table 2:** Primer pairs for RT-qPCR analysis.

Gene	Forward (F) and reverse (R) oligonucleotides	GenBank acc. no.	Amplicon size
*Actb*-F	ctaaggccaaccgtgaaag	NM_007393.5	104 bp
*Actb*-R	accagaggcatacagggaca		
*C1qa-*F	cgggtctcaaaggagagaga	NM_007572.2	71 bp
*C1qa-*R	tcctttaaaacctcggatacca		
*C1qb-*F	aggcactccagggataaagg	NM_009777.3	80 bp
*C1qb*-R	ggtcccctttctctccaaac		
*C1qc*-F	atggtcgttggacccagtt	NM_007574.2	75 bp
*C1qc*-R	gagtggtagggccagaagaa		
*C3*-F	accttacctcggcaagtttct	NM_009778.3	75 bp
*C3*-R	ttgtagagctgctggtcagg		
*Cd68*-F	tgatcttgctaggaccgctta	NM_001291058.1	66 bp
*Cd68*-R	taacggcctttttgtgagga		
*Cfb-*F	ctcgaacctgcagatccac	M57890.1	112 bp
*Cfb-*R	tcaaagtcctgcggtcgt		
*Gfap*-F	acagactttctccaacctccag	NM_010277.3	63 bp
*Gfap*-R	ccttctgacacggatttggt		
*Hc*-F	tgacaccaggcttcagaaagt	XM_017315669.2	69 bp
*Hc*-R	agttgcgcacagtcagctt		
*Iba1*-F	ggatttgcagggaggaaaa	D86382.1	92 bp
*Iba1*-R	tgggatcatcgaggaattg		
*Masp2*-F	ggcggctactattgctcct	NM_001003893.2	86 bp
*Masp2*-R	aacacctggcctgaacaaag		
*Pou4f1-F*	ctccctgagcacaagtaccc	AY706205.1	98 bp
*Pou4f1-R*	ctggcgaagaggttgctc		
*Ppid*-F	ttcttcataaccacaagtcaagacc	M60456.1	95 bp
*Ppid*-R	tccacctccgtaccacatc		
*Tgfb*-F	aggaggtttataaaatcgacatgc	XM_006497136.3	65 bp
*Tgfb*-R	tgtaacaactgggcagacagttt		
*Tmem119*-F	gtgtctaacaggccccagaa	NM_146162.3	110 bp
*Tmem119*-R	agccacgtggtatcaaggag		
*Tnfa*-F	ctgtagcccacgtcgtagc	NM_013693.3	97 bp
*Tnfa*-R	ttgagatccatgccgttg		

The primer pairs listed in the table were used in RT-qPCR experiments. Actin (Actb) and Cyclophilin (Ppid) served as housekeeping genes. F, forward; R, reverse.

### Statistical Analyses

Statistical analyses of clinical scores, SD-OCT, ERG, histology, and immunohistochemistry were performed using Statistica software (Version 13; StatSoft, Tulsa, OK, USA). Groups were compared using Student’s *t*-test for each time point. Data are shown as mean ± standard error of the mean (SEM). RT-qPCR data are shown as relative expression values with median ± quartile+minimum/maximum and were assessed *via* Pair Wise Fixed Reallocation Randomisation Test^©^ using REST^©^ software (Qiagen) (22). Incidence and the Spearman correlation coefficient (r, R^2^) were calculated for the analyses of the associations between SD-OCT parameters and RGC numbers as well as between clinical scores and RGC numbers/SD-OCT parameters using Prism (Version 9; GraphPad, San Diego, CA, USA). P<0.05 was considered as statistically significant, with *p<0.05, **p<0.01, and ***p<0.001.

## Results

### OSE Mice Develop Disability in Accordance With Reduced Weight

Single-transgenic control animals (IgH^MOG^) remained without neurological impairment (mean score 0) over the whole duration of the study, as expected. Contrary, OSE mice developed signs of spontaneous encephalomyelitis, starting at day 26 after birth with an incidence of 25% at day 27 (**Figures 1B, C**). After 32 days, 50% of OSE mice were affected as reflected in a mean score of 1.9 ± 1.0 (p=0.087). The incidence increased to 75% at day 38 in OSE mice (2.3 ± 1.0; p=0.033; **Figure 1C**), reflecting a flaccid tail and an impaired righting reflex or gait. At day 56, the mean EAE score in the OSE group was 4.3 ± 0.8.

Disability in OSE mice was reflected in a 10.8% lower weight (14.1 ± 1.1 g) compared to control mice after six weeks (15.8 ± 0.6 g, p=0.199). Eight-week-old OSE animals weighted significantly less (15.0 ± 1.0 g) than the control group (19.3 ± 0.6 g, p=0.003; **Supplemental Figure 1**).

### Retinal Thickness Is Reduced in OSE

To evaluate structural alterations of the retina, we performed SD-OCT after six and eight weeks (**Figure 1D**). The analysis in six weeks old mice showed a significant reduction of the retinal thickness by 8.2% in OSE retinae (345.3 ± 4.2 µm) compared to the control group (376.1 ± 2.5 µm; p<0.001; **Figure 1E**). To investigate whether there was loss of retinal thickness over time, we also performed analyses at eight weeks of age. We found a reduced thickness of 12.3% in OSE animals (314.6 ± 3.1 µm) compared to the control group (358.9 ± 3.1 µm; p<0.001). In addition, we noted a progressive thinning of retina in OSE mice from six to eight weeks (8.9%, p<0.001; **Figure 1E**).

To analyze which layers are affected by the reduction of retinal thickness, separate measurements of the ganglion cell complex (=RNFL, GCL and IPL), the INL, and the ONL were carried out. At 6 weeks, OSE mice had a thinner ganglion cell complex (control: 151.9 ± 0.8 µm, OSE: 133.5 ± 1.8 µm, p<0.001). We found a thinning by 6.5% of the ganglion cell complex in the OSE groups at eight weeks (control: 150.2 ± 1.1 µm, OSE: 124.7 ± 1.9 µm, p<0.001; **Figure 1F**).

A significantly reduced INL (control: 45.6 ± 4.2 µm, OSE: 40.7 ± 1.0 µm, p=0.016; **Figure 1G**) and ONL thickness (control: 132.4 ± 0.7 µm, OSE: 122.4 ± 2.5 µm, p=0.002; **Figure 1H**) was noted at the six weeks´ time point. The INL in the OSE group was also thinner at eight weeks (control: 45.1 ± 0.8 µm, OSE: 39.7 ± 0.3 µm, p<0.001; **Figure 1G**). The ONL thickness at eight weeks was also reduced in the OSE group (control: 131.6 ± 1.5 µm, OSE: 121.9 ± 1.6 µm, p<0.001; **Figure 1H**).

### Reduction of Retinal Electrical Output as Marker of Impaired Function

**Figure 2:**
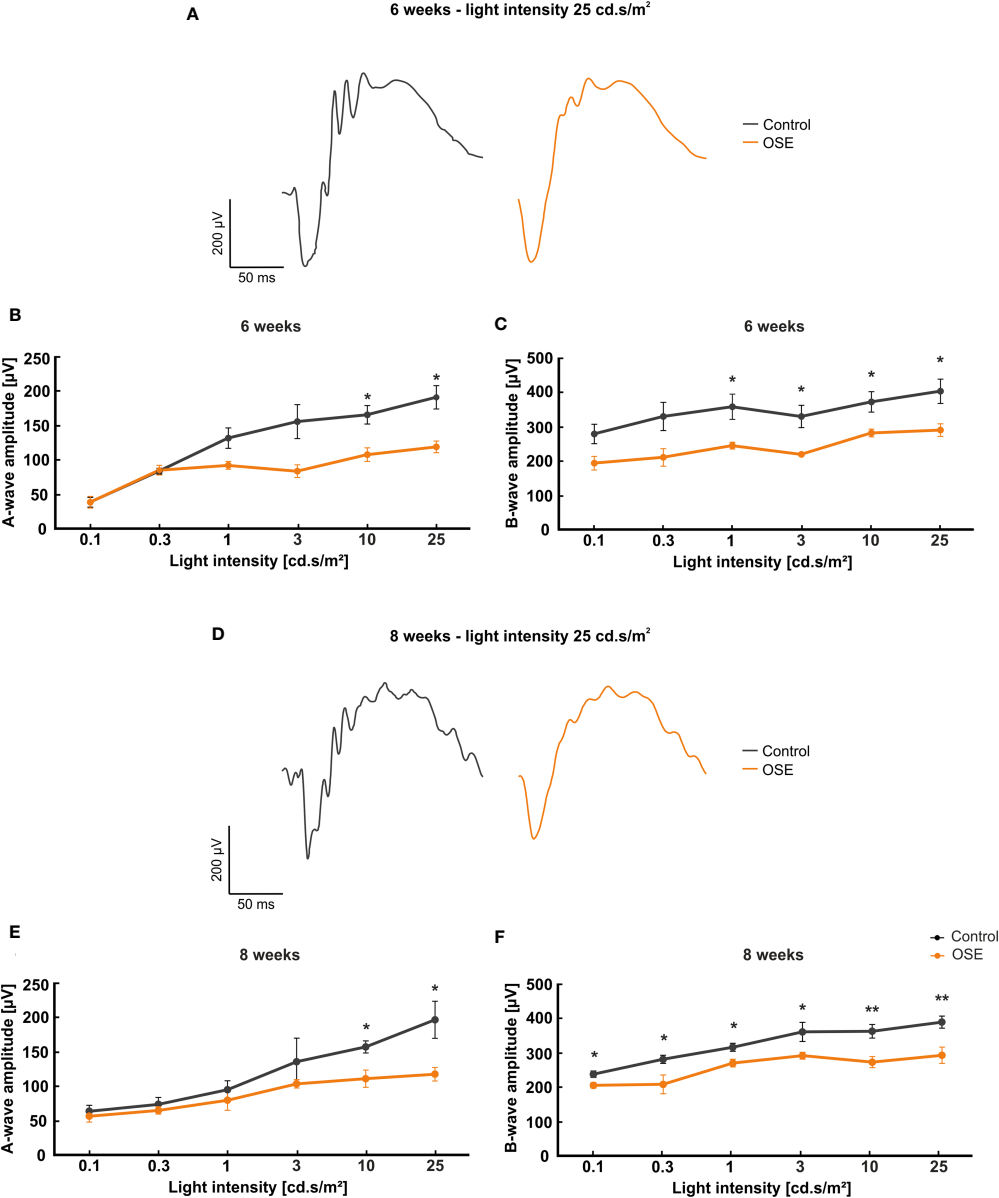
Functional impairment of the retina. **(A)** Exemplary ERG amplitudes at 25 cd.s/m^2^ flash luminance at six weeks. **(B)** At six weeks, the a-wave amplitude, representing the photoreceptors, was reduced in the OSE group. **(C)** The b-wave amplitude, which illustrates the conductivity of the inner retinal layers, was significantly reduced in OSE animals in comparison to the control group. **(D)** Exemplary ERG amplitudes at a flash luminance of 25 cd.s/m^2^ in eight-week-old mice. **(E)** The ERG measurements after eight weeks again displayed a reduced a-wave amplitude in OSE. **(F)** Also, a loss of the electrical output was noted after eight weeks regarding the b-wave amplitude of OSE mice. Data are shown as mean ± SEM. *p < 0.05, **p < 0.01.

To understand whether loss of retinal thickness is also associated with functional impairment, we performed ERG recordings. In all groups, increased a- and b-wave amplitudes were observed with rising light flash stimuli. At six and eight weeks, ERG waveforms at a flash luminance of 25 cd*s/m^2^ showed a decrease in OSE animals compared to controls (**Figure 2A, D**).

The a-wave represents the electrical output of the photoreceptor layer and was significantly reduced at flash intensities of 10 and 25 cd s/m^2^ in OSE mice after six weeks (p=0.012 at 10 cd.s/m^2^, p=0.032 at 25 cd.s/m^2^). At 0.1 and 0.3 cd.s/m^2^ flash intensity, the a-wave amplitude of the OSE group was similar to the one of the control group. A non-significant reduction of the a-wave amplitude could be noted at 1 (p=0.059) and 3 cd.s/m^2^ (p=0.087) flash intensity (**Figure 2B**).

The b-wave mirrors the electrical conductivity of the inner retinal layers. A non-significant trend for a decreased b-wave amplitude was measured at flash intensities of 0.1 (p=0.051) and 0.3 cd.s/m^2^ (p=0.057) in OSE mice in comparison to the control group at six weeks. A significant reduction could be documented at flash intensities of 1 (p=0.034) up to 25 cd.s/m^2^ (p=0.03) in OSE mice (**Figure 2C**).

The ERG measurements after eight weeks showed a non-significant reduction of the a-wave amplitude from 0.1 (p=0.566) to 3 cd.s/m^2^ (p=0.377). At flash intensities of 10 and 25 cd.s/m^2^, a significantly decreased a-wave amplitude was measurable in OSE mice (p=0.013 at 10 cd.s/m^2^, p=0.02 at 25 cd.s/m^2^; **Figure 2E**). The b-wave amplitude was strongly decreased at all flash intensities in OSE mice after eight weeks (p=0.024 at 0.1 cd.s/m^2^, p=0.034 at 0.3 cd.s/m^2^, p=0.017 at 1 cd.s/m^2^, p=0.04 at 3 cd.s/m^2^, p=0.006 at 10 cd.s/m^2^, p=0.008 at 25 cd.s/m^2^; **Figure 2F**). Those data altogether indicate an impairment of the retinal function, which might be explained by a dysfunction of photoreceptor and bipolar cells in affected mice.

### Cellular Infiltration, Inflammation, and Demyelination of Optic Nerves in OSE

**Figure 3:**
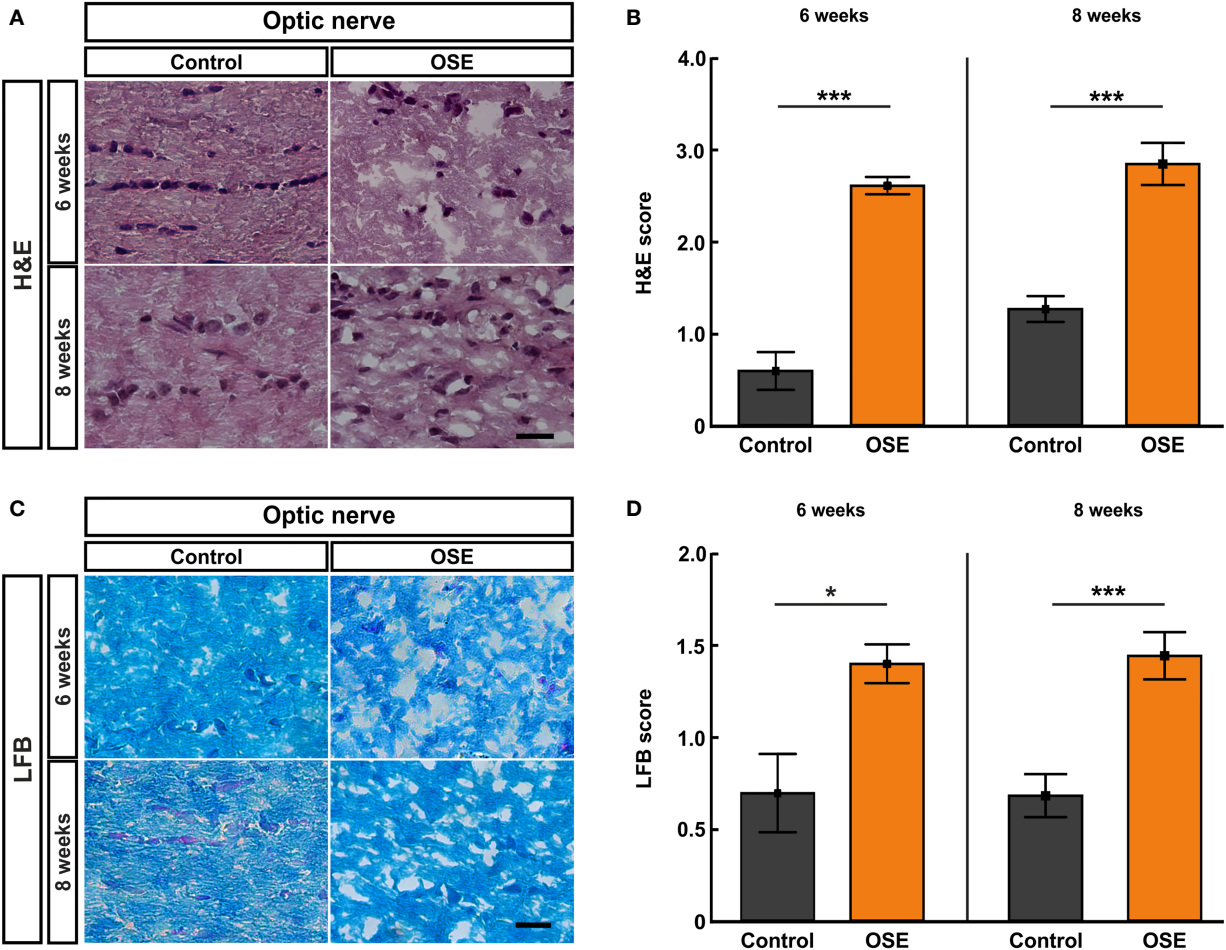
Structural damage and increased demyelination of the optic nerve. **(A)** H&E stainings were performed after six and eight weeks to evaluate the morphology of the optic nerve. **(B)** The optic nerves of OSE mice showed a significantly elevated H&E score compared to the control group at both ages. **(C)** LFB-stained myelin sheaths of the control and OSE group are displayed. **(D)** Demyelination was increased in the OSE group as indicated in a higher LFB score at six as well at eight weeks of age. Data are shown as mean ± SEM. *p < 0.05, ***p < 0.001. Scale bars: 20 μm.

We performed histological analyses of optic nerves to investigate the degree of inflammation and demyelination (19). The H&E staining of optic nerve sections was evaluated via scoring the degree of cellular infiltration (**Figure 3A**). After six weeks, OSE mice showed an average score of 2.6 ± 0.1, which was increased compared to the control group (0.6 ± 0.2, p<0.001). The H&E score in the control group increased from six to eight weeks (1.3 ± 0.1; p=0.02). Still, more cellular infiltration and inflammation was also notable in H&E stained OSE nerves at eight weeks of age (2.8 ± 0.2), when compared to controls (p<0.001 **Figure 3B**).

Histopathological stainings with LFB were performed to investigate the degree of demyelination after six and eight weeks (**Figure 3C**). LFB-stained myelin sheaths of the six-week-old control group resembled combed bundles in a parallel arrangement (0.7 ± 0.2). This arrangement was interrupted in the OSE group represented by the brightening of the structure and demyelination (1.4 ± 0.1; p=0.011). At eight weeks of age, demyelination was again significantly higher in the OSE group (1.4 ± 0.1) compared to the control (0.7 ± 0.1; p<0.001; **Figure 3D**).

### Increased Infiltration of Activated Microglia in the Optic Nerve of OSE Mice

**Figure 4:**
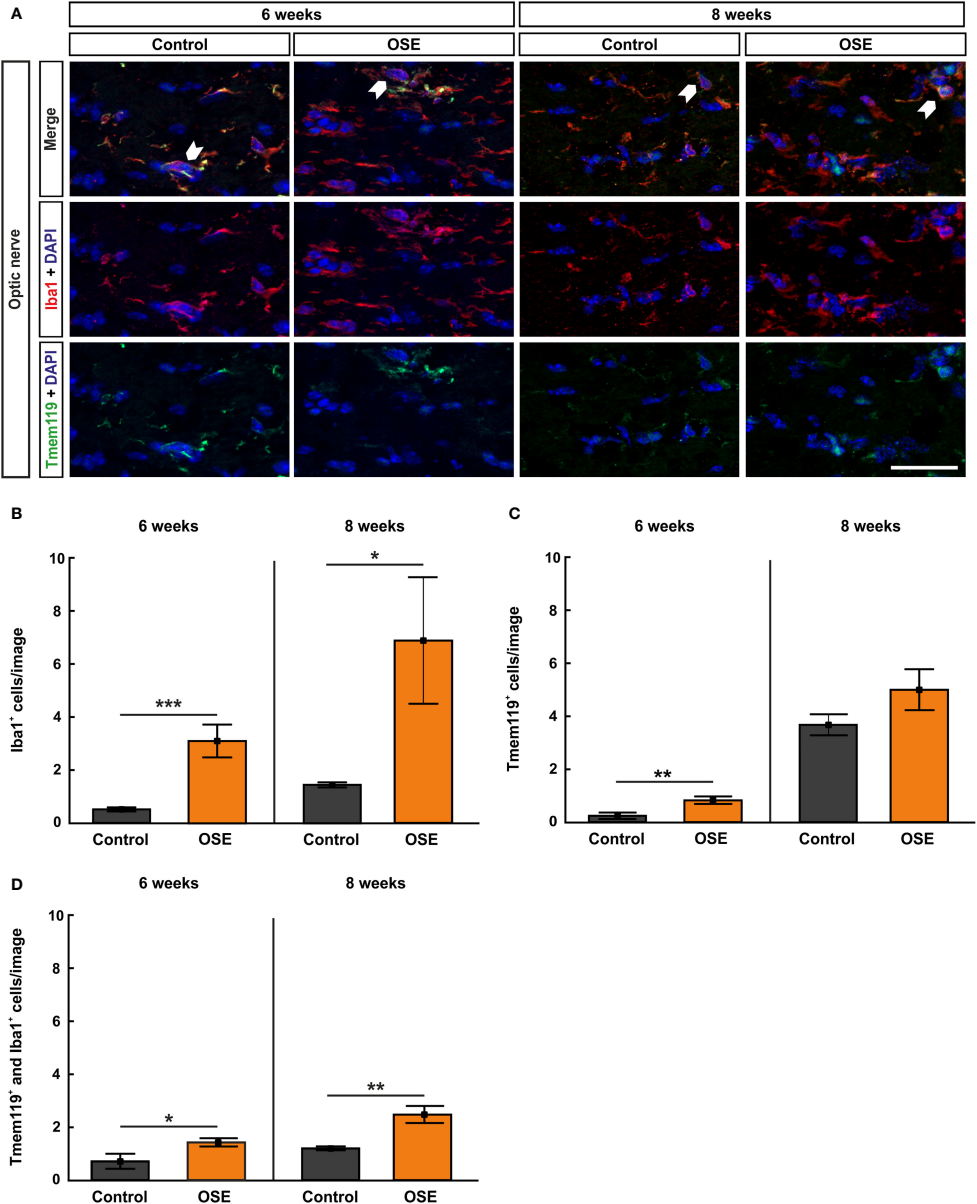
Increased numbers of microglia/macrophages in the optic nerve. **(A)** Optic nerve sections were stained with Iba1 (red, microglia/macrophages) and Tmem119 (green, microglia) at six and after eight weeks. White arrows indicate co-localized Tmem119^+^ and Iba1^+^ cells. **(B)** The number Iba1^+^ cells was significantly increased in the OSE group after six weeks. The staining after eight weeks still displayed an increase of Iba1^+^ cells among the OSE group. **(C)** Tmem119^+^ cells were also increased after six weeks. No difference of the number of Tmem119^+^ cells was measurable between both groups at eight weeks. **(D)** A higher number of microglia (Tmem119^+^ and Iba1^+^) was observed at six weeks. This elevation was still noted in the OSE group at eight weeks. Data are shown as mean ± SEM. *p < 0.05, **p < 0.01, ***p < 0.001. Scale bar: 20 μm.

Iba1 was used to label microglia/macrophages (23, 24). Co-staining with Tmem119, the transmembrane protein which is exclusively expressed by microglia, was used to visualize microglia on optic nerve sections (25) (**Figure 4A**). The results at six weeks display a significantly higher number of Iba1^+^ cells in the OSE group (3.1 ± 0.6 cells) in comparison to controls (0.5 ± 0.1 cells, p<0.001). At eight weeks, Iba1^+^ cell numbers were still increased in OSE mice (6.9 ± 2.4 cells) compared to controls (1.4 ± 0.1 cells, p=0.042; **Figure 4B**).

Tmem119^+^ cells showed a threefold increase in six-week-old OSE mice (0.8 ± 0.1 cells) compared to controls (0.2 ± 0.1 cells, p=0.007). At eight weeks, the mean Tmem119 cell count in the control group (3.7 ± 0.4 cells) was higher than in younger animals (p<0.001). The number of Tmem119^+^ cells was similar in the OSE group (5.0 ± 0.8 cells) and the control group at eight weeks (p=0.152; **Figure 4C**).

In addition, a twofold increase in Tmem119^+^ and Iba1^+^ cells was noted in OSE mice at the age of six weeks (1.4 ± 0.2 cells) compared to controls (0.7 ± 0.3 cells, p=0.045). The number of Tmem119^+^and Iba1^+^ cells was still significantly increased in OSE (2.5 ± 0.3 cells) at eight weeks (control: 1.2 ± 0.1 cells, p=0.002; **Figure 4D**).

### OSE Mice Display a Reduced Number of Retinal Ganglion Cells

**Figure 5:**
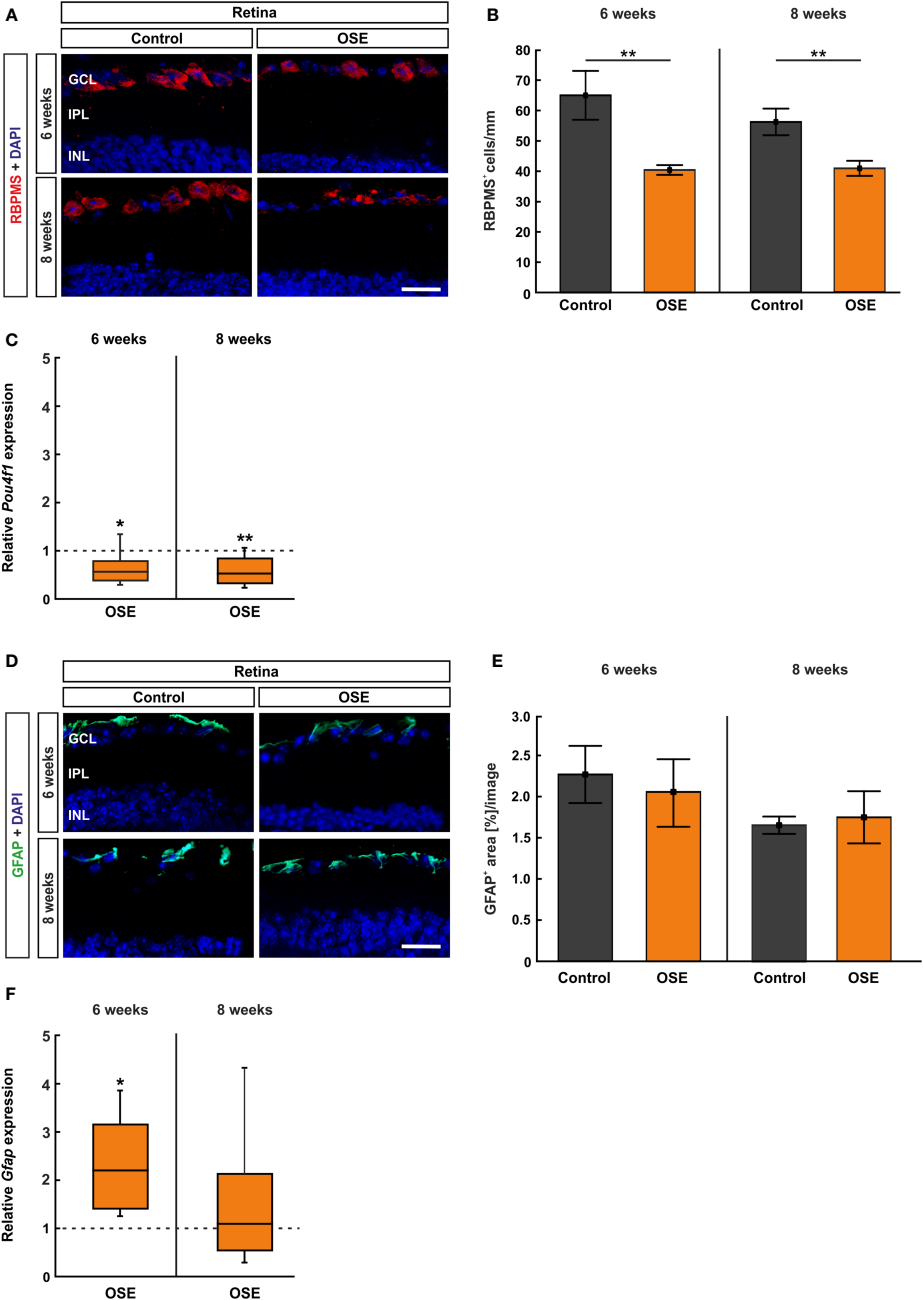
Reduced number of retinal ganglion cells in OSE mice. **(A)** Retinal ganglion cells were stained with RBPMS (red) on retinal cross-sections. Cell nuclei were labeled with DAPI (blue). **(B)** The number of retinal ganglion cells was significantly reduced in the OSE group at six weeks. This reduction was also detectable in eight-week-old animals. **(C)** A significant downregulation of *Pou4f1* mRNA was noted in OSE mice in comparison to the control group at six and eight weeks. **(D)** Immunohistological staining of retinal cross-sections with GFAP (green) and DAPI (blue) was performed to label macroglia. **(E)** The GFAP^+^ area evaluation revealed no significant differences between both groups at both ages. **(F)** The expression of *Gfap* mRNA was significantly increased in OSE mice at six weeks, but no changes were detectable at eight weeks. Data are mean ± SEM for immunohistochemistry, relative values for RT-qPCR are median ± quartile ± maximum/minimum. The dotted lines in **(C, F)** represent the relative expression level of the control group. GCL, ganglion cell layer; IPL, inner plexiform layer; INL, inner nuclear layer. *p < 0.05, **p < 0.01. Scale bars: 20 μm.

We used RBPMS to label RGCs (26) (**Figure 5A**). After six weeks, the number of RGCs was reduced by 37.8% in OSE mice (40.4 ± 1.6 cells/mm) in comparison to the control group (65.0 ± 8.1 cells/mm, p=0.01). A reduction of RGCs by 27.4% was noted in eight-week-old OSE mice (OSE: 42.7 ± 2.6 cells/mm; control: 58.8 ± 4.6 cells/mm, p=0.007; **Figure 5B**).

Additionally, we quantified the *Pou4f1* expression, which is a transcription factor highly expressed in RGCs, using RT-qPCR. In conformity with the immunohistochemical staining, the RT-qPCR displayed a significantly lower *Pou4f1* mRNA expression in the OSE group after six weeks (0.563-fold, p=0.015) in comparison to the control group. The RT-qPCR analyses of the eight-week time point showed a similar result (0.519-fold, p=0.008; **Figure 5C**).

The immunohistological staining with GFAP to analyze macroglia was performed after six and eight weeks (**Figure 5D**), since retinal degeneration is often associated with gliosis (27). The analysis of the GFAP^+^ area displayed no significant differences between both groups at six (OSE: 2.1 ± 0.4%, control: 2.3 ± 0.3%, p=0.702) and at eight weeks (OSE: 1.7 ± 0.3%, control: 1.7 ± 0.1%, p=0.795; **Figure 5E**). Interestingly, an increased *Gfap* mRNA expression was detectable at six weeks (2.193-fold, p=0.005) whereas no significant changes were notable after eight weeks (1.087-fold, p=0.81, **Figure 5F**).

### Increased Number of Microglia/Macrophages in the Retina

**Figure 6:**
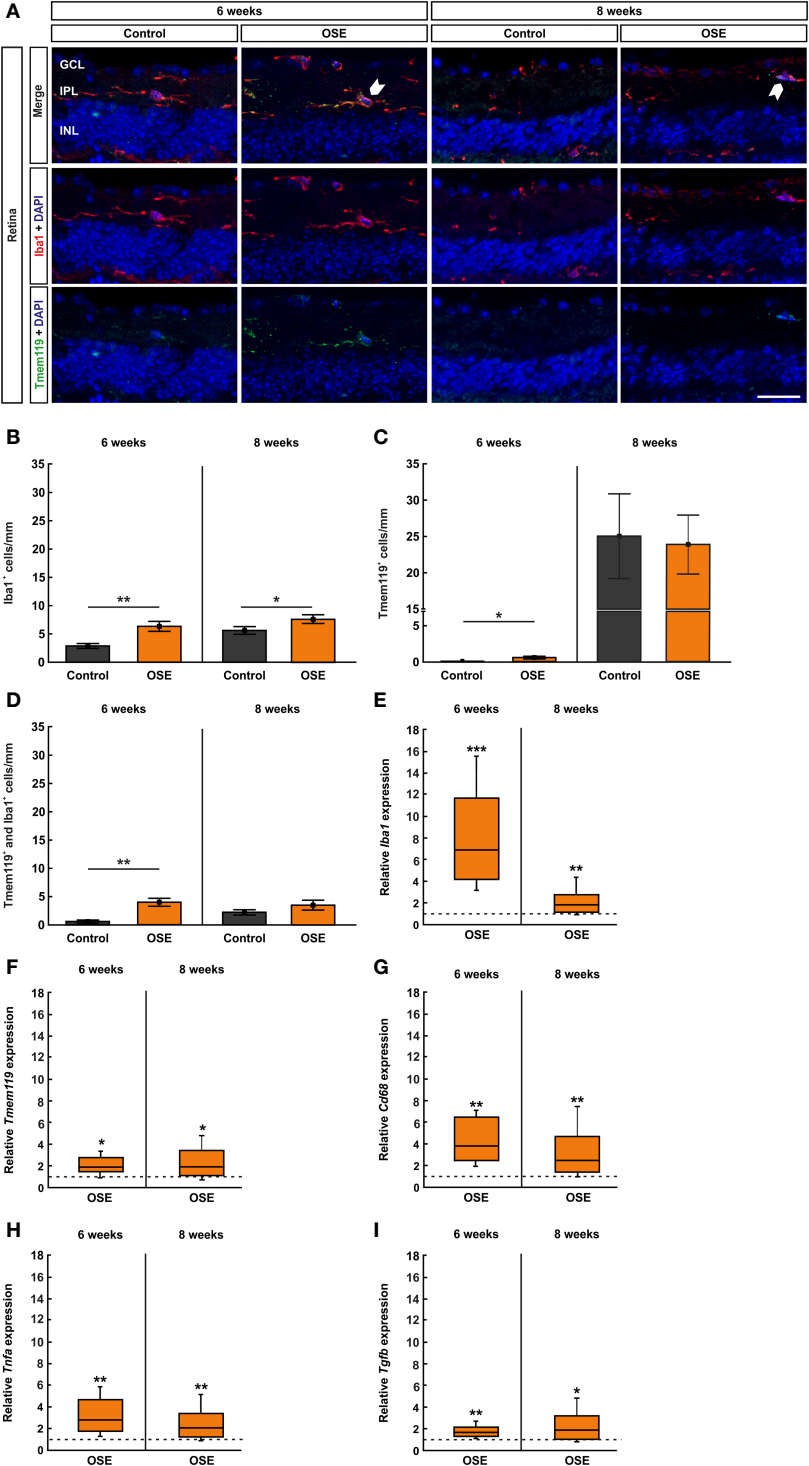
Increased number of microglia/macrophages in OSE retinae. **(A)** Iba1 was used to label microglia/macrophages (red) whereas microglia were co-stained with Tmem119 (green) and Iba1. Iba1^+^ and Tmem119^+^ cells were quantified in GCL, IPL, and INL. White arrows point towards co-localized Tmem119^+^ and Iba1^+^ cells. **(B)** The immunohistochemical staining of the retina after six weeks demonstrated a significantly increased number of microglia/macrophages (Iba1^+^) in OSE mice. The number of Iba1^+^ cells was also significantly increased in eight-week-old OSE mice. **(C)** An increased number of Tmem119^+^ cells was noted in the retinae of OSE animals at six weeks. Tmem119^+^ cell counts were comparable in both groups at eight weeks. **(D)** The number of Tmem119^+^ and Iba1^+^ cells in the retina was significantly increased in the OSE group at six weeks, which was diminished at eight weeks. **(E)** Using RT-qPCR, a significant upregulation of *Iba1* mRNA was noticed in six-week-old OSE mice. After eight weeks, the expression of *Iba1* mRNA was still significantly increased, though to a lesser extent. **(F)** The expression of *Cd68* mRNA was significantly increased in OSE retinas at both time points. **(G)** The mRNA expression of *Tmem119* was also elevated after six and eight weeks. **(H)** RT-qPCR evaluation of the cytokine *Tnfa* mRNA revealed an increased expression at both ages in OSE mice. **(I)** The *Tgfb* mRNA expression was significantly upregulated at six and eight weeks in the OSE group. Data are shown as mean ± SEM for immunohistochemistry, relative values for RT-qPCR are median ± quartile ± maximum/minimum. The dotted lines in **(E–I)** represent the relative expression level of the control group. GCL, ganglion cell layer; IPL, inner plexiform layer; INL, inner nuclear layer. *p < 0.05, **p < 0.01, ***p < 0.001. Scale bar: 20 μm.

The staining with Iba1 and Tmem119 was likewise performed on retinal cross-sections (**Figure 6A**) and cells were counted in the inner retinal layers (GCL to INL) together as well as separately. The immunohistochemical staining of the retina after six weeks demonstrated a significantly increased number of microglia/macrophages (Iba1^+^ cells) in OSE mice (6.4 ± 0.9 cells/mm) compared to the control group (2.9 ± 0.4 cells/mm, p=0.005). After eight weeks, the number of Iba1^+^ cells was still significantly increased in OSE animals (7.6 ± 0.8 cells/mm) compared to controls (5.5 ± 0.6 cells/mm, p=0.048, **Figure 6B**). Regarding the separate layers, at six weeks, more Iba1^+^ cells were noted in the GCL of OSE mice (3.3 ± 0.7 cells/mm) compared to controls (0.4 ± 0.2 cells/mm, p=0.002), but not at eight weeks (OSE: 2.7 ± 0.4%, control: 1.9 ± 0.4%, p=0.141; **Supplemental Figure 2A**). The number of Iba1^+^ cells was comparable in both groups in the IPL at six (OSE: 2.4 ± 0.3%, control: 1.4 ± 0.9%, p=0.054) and eight weeks (OSE: 1.3 ± 0.4%, control: 0.7 ± 0.1%, p=0.146; **Supplementary Figure 2B**). Also, no difference between both groups was observed regarding Iba1^+^ cell counts in the INL at six (OSE: 0.7 ± 0.3%, control: 1.1 ± 0.3%, p=0.187) and eight weeks (OSE: 3.6 ± 0.6%, control: 3.0 ± 0.6%, p=0.469; **Supplemental Figure 2C**).

Additionally, we noted an increase of Tmem119^+^ cells in the retinae of OSE animals after six weeks (OSE: 0.6 ± 0.2 cells/mm; control: 0.05 ± 0.05 cells/mm, p=0.028). No significant differences regarding Tmem119^+^ cells were detectable at eight weeks of age (OSE: 23.9 ± 4.0 cells/mm, control: 23.0 ± 5.4 cells/mm, p=0.869; **Figure 6C**). When examining Tmem119^+^ cells in the retinal layers separately, no differences between the groups could be detected. The number of Tmem119^+^ cells was similar in the GCL in OSE and control retinas at six (OSE: 2.4 ± 0.3%, control: 1.4 ± 0.9%, p=0.105) and eight weeks (OSE: 1.7 ± 0.5%, control: 1.9 ± 0.5%, p=0.775; **Supplemental Figure 2D**). Also, Tmem119^+^ cell counts were comparable in the IPL at six (OSE: 0.1 ± 0.1%, control: 0.0 ± 0.0%, p=0.079) and eight weeks (OSE: 1.0 ± 0.2%, control: 0.8 ± 0.3%, p=0.585; **Supplemental Figure 2E**). The same was the case in the INL at six (OSE: 0.3 ± 0.5%, control: 0.06 ± 0.1%, p=0.927) and eight weeks (OSE: 21.2 ± 4.0%, control: 20.7 ± 4.8%, p=0.775; **Supplemental Figure 2F**).

The co-staining of Tmem119 and Iba1 showed significantly more microglia in the retinas of six-week-old OSE mice (4.0 ± 0.7 cells/mm) in comparison to the controls (0.6 ± 0.3 cells/mm, p=0.001). In contrast, no significant changes were detectable at eight weeks (OSE: 3.5 ± 0.9 cells/mm, control: 2.1 ± 0.4 cells/mm, p=0.229; **Figure 6D**). In regard to the cell counts in the separate layers, significant differences were only visible in six-week-old mice. Here, OSE mice (0.7 ± 0.2%) displayed more Tmem119^+^ and Iba1^+^ cells in the GCL than control animals (0.1 ± 0.1%, p=0.005). Later on, cell counts were comparable in these groups (OSE: 0.7 ± 0.3%, control: 0.4 ± 0.2%, p=0.336; **Supplemental Figure 2G**). The Tmem119^+^ and Iba1^+^ cell numbers in the IPL were higher in OSE mice (2.1 ± 0.4%) than in controls (0.5 ± 0.2%, p=0.003) at six weeks, while at eight weeks, cell counts in this layer were similar in both groups (OSE: 0.6 ± 0.3%, control: 0.2 ± 0.3%, p=0.206; **Supplemental Figure 2H**). In addition, in the INL more, Tmem119^+^ and Iba1^+^ cells were counted in the OSE group (4.0 ± 0.7%) than in the control group (2.0 ± 0.7%, p=0.001). At eight weeks, cell counts in both groups in the INL were not different (OSE: 3.5 ± 0.9%, control: 2.1 ± 0.9%, p=0.156; **Supplemental Figure 2I**).

In addition to the immunhistochemical stainings, we also performed RT-qPCR to quantify the expression of the markers *Iba1* (microglia/macrophages), *Tmem119* (microglia), and *CD68* (ED1, macrophages). The analyses via RT-qPCR at six weeks showed a marked increase of the *Iba1* mRNA expression in retinae of OSE mice (6.95-fold, p<0.001). After eight weeks, the expression of *Iba1* mRNA was still increased, though to a lesser extent (1.803-fold, p=0.008; **Figure 6E**). Additionally, we found an increased *Tmem119* mRNA expression in OSE in six- (1.888-fold, p=0.026) and eight-week-old OSE mice (1.963-fold, p=0.016; **Figure 6F**). The expression of *CD68* mRNA was also higher in the OSE group at six weeks (3.877-fold, p=0.002) as well as at eight weeks of age (2.469-fold, p=0.007; **Figure 6G**).

Since microglia are known to be the primary source of proinflammatory cytokines, we analyzed the mRNA expression of *Tnfa* and *Tgfb*. We noted increased mRNA expression levels at both time points regarding *Tnfa* (6 weeks: 2.813-fold, p=0.005; 8 weeks: 2.071-fold, p=0.006; **Figure 6H**). The mRNA expression of *Tgfb* was also upregulated in OSE mice after six (1.682-fold, p=0.003) as well as after eight weeks (1.899-fold, p=0.023, **Figure 6I**).

### Activation of the Complement System in the Retina of OSE Mice

**Figure 7:**
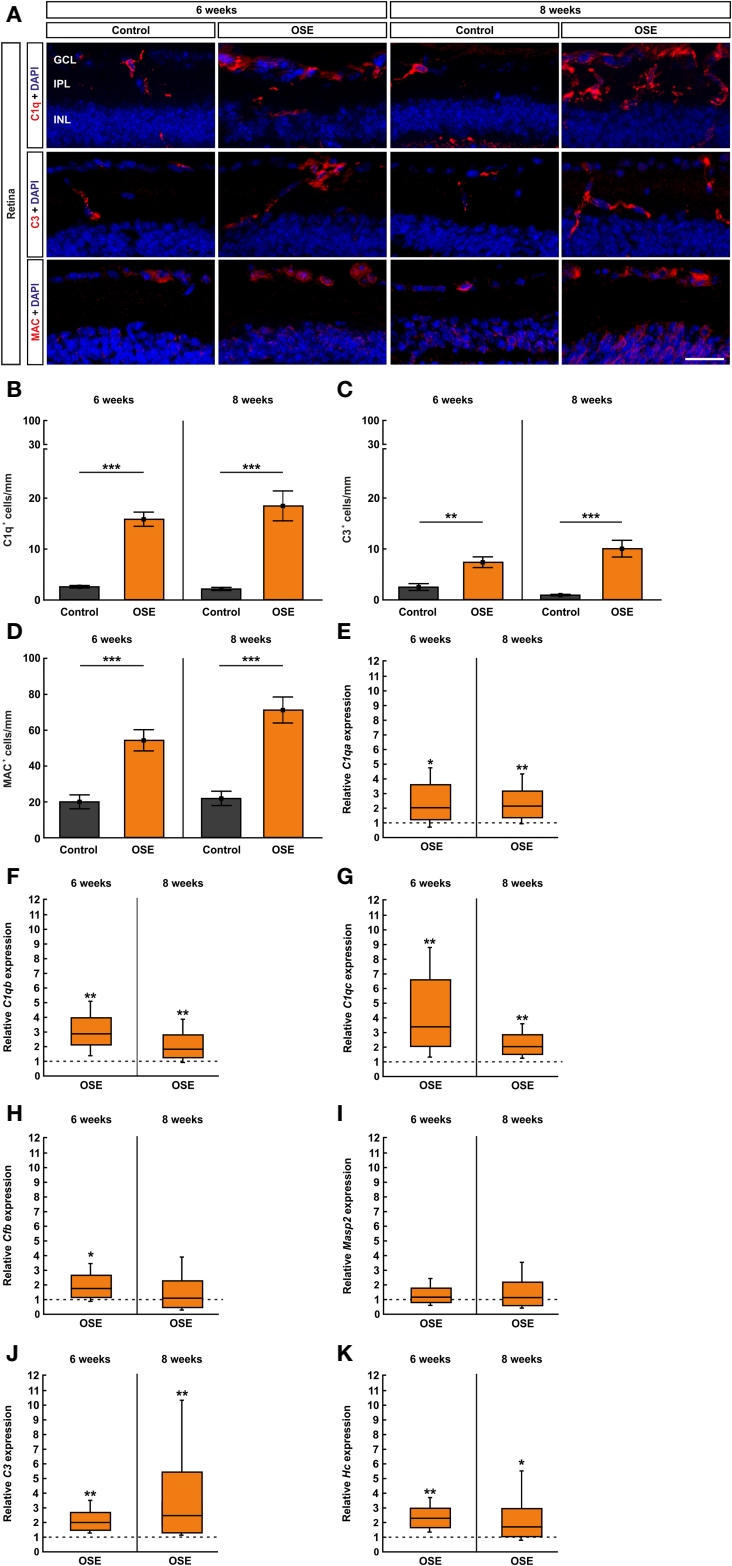
Activation of the classical pathway of the complement system in the retina of OSE mice. **(A)** Staining of retinal cross-sections with the complement markers C1q (red), C3 (red), and MAC (red), while DAPI counterstained cell nuclei (blue). **(B)** A significantly increased number of C1q^+^ cells was found in the OSE group in the GCL, IPL, and INL after six weeks. An increase of C1q^+^ cells of 16.5% was noted at the eight weeks´ time point. **(C)** The total number of C3^+^ cells in GCL, IPL, and INL was significantly elevated in six-week-old OSE mice. After eight weeks, C3^+^ cells were even further increased. **(D)** The quantification of MAC^+^ cells revealed a significant increase among the OSE group at six weeks. A progression of increasing MAC^+^ cells in OSE mice was observed after eight weeks. **(E–G)**. Complement pathway factor *C1qa*, *C1qb*, and *C1qc* (classical pathway) mRNA expression were significantly upregulated in six- and eight-week-old OSE mice. **(H)** At six weeks of age, a significant increase of *Cfb* (Factor B, alternative pathway) mRNA expression was noted in OSE retinae, whereas the expression was not significantly altered later on, at eight weeks. **(I)** No significant differences were found in *Masp2* mRNA (lectin pathway) at six and eight weeks. **(J)** An increased *C3* (common pathway) mRNA expression was detected in the OSE group at both ages. **(K)** Likewise, the mRNA level of *Hc* (C5, common pathway) was upregulated at six as well as at eight weeks in OSE retinae. Data are shown as mean ± SEM for immunohistochemistry, relative values for RT-qPCR are median ± quartile ± maximum/minimum. The dotted lines in **(E–K)** represent the relative expression level of the control group. GCL, ganglion cell layer; IPL, inner plexiform layer; INL, inner nuclear layer. *p < 0.05, **p < 0.01, ***p < 0.001. Scale bar: 20 μm.

Retinal cross-sections were labelled with antibodies against the complement markers C1q, C3, and MAC (**Figure 7A**) and counted in the inner retinal layers (GCL to INL). The staining with C1q at the six weeks´ time point showed a significantly higher number of C1q^+^ cells in the OSE group (15.8 ± 1.4 cells/mm) in comparison to the control group (2.6 ± 0.3 cells/mm, p<0.001). An increase of 16.5% among C1q^+^ cells was noted in eight-week-old OSE mice (OSE: 18.4 ± 3.0 cells/mm; control: 2.1 ± 0.3 cells/mm, p<0.001; **Figure 7B**). When looking at the retinal layers separately, at six weeks, we noted a more than 10-fold increase of C1q^+^ cells in the GCL of OSE animals (7.1 ± 0.9 cells/mm) compared to controls (0.6 ± 0.2 cells/mm, p<0.001). At eight weeks, OSE animals (6.8 ± 1.3 cells/mm) still showed more C1q^+^ cells in the GCL than controls (0.7 ± 0.2 cells/mm, p<0.001; **Supplemental Figure 3A**). Regarding the IPL, the OSE group (6.0 ± 0.8 cells/mm) displayed more C1q^+^ cells than controls (1.4 ± 0.3 cells/mm, p<0.001) at six weeks. Similar effects were noted at eight weeks (OSE: 9.5 ± 1.4 cells/mm; control: 1.0 ± 0.2 cells/mm, p<0.001; **Supplemental Figure 3B**). More C1q^+^ cells were also noted in the INL of OSE retinae at six (OSE: 2.7 ± 0.5 cells/mm; control: 0.5 ± 0.2 cells/mm, p=0.001) and eight weeks (OSE: 2.1 ± 0.4 cells/mm; control: 0.4 ± 0.1 cells/mm, p=0.002; **Supplemental Figure 3C**).

The complement marker C3 was stained on retinal cross-sections (**Figure 7A**). The statistical analysis showed a significant increase of C3^+^ cells in GCL, IPL, and INL together in the OSE group (7.3 ± 1.1 cells/mm) compared to the control (2.4 ± 0.7 cells/mm, p=0.003) after six weeks. An increase of C3^+^ cells in GCL, IPL, and INL was notable in eight-week-old OSE mice (10.0 ± 1.7 cells/mm; control: 0.9 ± 0.2 cells/mm, p<0.001; **Figure 7C**). We also investigated the number of C3^+^ cells in these layers separately. We mainly found an upregulation in the OSE group. The number of C3^+^ cells in the GCL was higher in OSE retinae at six (OSE: 3.5 ± 0.6 cells/mm; control: 0.9 ± 0.4 cells/mm, p=0.003) and eight weeks (OSE: 2.4 ± 0.9 cells/mm; control: 0.04 ± 0.04 cells/mm, p=0.0147; **Supplemental Figure 3D**). In the IPL, C3^+^ cell counts were also elevated in OSE animals at six (OSE: 1.7 ± 0.4 cells/mm; control: 0.8 ± 0.2 cells/mm, p=0.050) and eight weeks (OSE: 2.5 ± 0.4 cells/mm; control: 0.4 ± 0.1 cells/mm, p<0.001; **Supplemental Figure 3E**). C3^+^ cell counts in the INL were similar in both groups at six weeks (OSE: 2.1 ± 0.6 cells/mm; control: 0.8 ± 0.4 cells/mm, p=0.131). At eight weeks, C3^+^ cell counts were higher in the INL of OSE animals (5.1 ± 0.8 cells/mm) than in controls (0.5 ± 0.2 cells/mm, p<0.001; **Supplemental Figure 3F**).

To complete the immunohistological analyses of the complement system, we used an antibody to label the terminal complex MAC (**Figure 7A**). The results of the six weeks´ time point showed an increase of MAC^+^ cells in the retinae of OSE mice (54.2 ± 6.0 cells/mm) compared to the control group (19.8 ± 3.9 cells/mm, p<0.001). The number of MAC^+^ cells increased by 31.0% in eight-week-old OSE animals (71.0 ± 7.3 cells/mm; control: 21.7 ± 4.1 cells/mm, p<0.001; **Figure 7D**). In the GCL of six-week-old OSE mice (23.5 ± 3.3 cells/mm) more MAC^+^ cells were noted than in controls (11.8 ± 2.9 cells/mm, p=0.020). At eight weeks, counts in both groups were comparable (OSE: 16.1 ± 3.1 cells/mm; control: 10.9 ± 2.4 cells/mm, p=0.208; **Supplemental Figure 3G**). MAC^+^ cell numbers were comparable in the IPL of both groups at six (OSE: 0.1 ± 0.1 cells/mm; control: 0.05 ± 0.05 cells/mm, p=0.403) and eight weeks (OSE: 0.0 ± 0.0 cells/mm; control: 0.05 ± 0.05 cells/mm, p=0.337; **Supplemental Figure 3H**). In the INL, the number of MAC^+^ cells was significantly higher in the OSE group at six (OSE: 30.6 ± 5.3 cells/mm; control: 8.0 ± 3.1 cells/mm, p=0.003) and eight weeks (OSE: 54.9 ± 7.9 cells/mm; control: 11.5 ± 4.9 cells/mm, p<0.001; **Supplemental Figure 3I**).

In addition, we performed RT-qPCR to quantify the mRNA expression of the complement pathway factors *C1qa*, *C1qb*, *C1qc*, *C3, Hc* (C5)*, Masp2* (mannan-binding lectin serine protease 2), *and Cfb* (Factor B). To investigate an involvement of the classical pathway, we analyzed the mRNA levels of *C1qa*, *C1qb*, and *C1qc*. The expression of *C1qa* mRNA was significantly upregulated in six-week-old OSE mice (2.036-fold, p=0.049). At eight weeks, an increase of the *C1qa* mRNA expression was still notable in the OSE group (2.147-fold, p=0.007; **Figure 7E**). The analyses of *C1qb* mRNA showed a significantly increased expression in OSE animals at six weeks (2.892-fold, p=0.004) as well as at eight weeks (1.847-fold, p=0.007; **Figure 7F**). In accordance with the upregulation of *C1qa* and *C1qb*, the expression of *C1qc* mRNA was also upregulated in OSE mice with six weeks of age (3.387-fold, p=0.008). Again, we found an increased expression of *C1qc* mRNA at eight weeks (2.04-fold, p=0.001; **Figure 7G**).

To analyze a potential involvement of the alternative pathway, we quantified the expression of *Cfb* mRNA which encodes Factor B. Indeed, we found a significant upregulation in six-week-old OSE animals in comparison to the control group (1.733-fold, p=0.021). In contrast, we noted no significant alteration of *Cfb* mRNA expression later on, at eight weeks of age (1.077-fold, p=0.834; **Figure 7H**).

The lectin pathway was evaluated via *Masp2*. *Masp2* mRNA expression was similar in both groups at six weeks (1.168-fold, p=0.468) and eight weeks (1.142-fold, p=0.665; **Figure 7I**), indicating that the lectin pathway is not activated in OSE mice.

To evaluate the common pathway which leads to the formation of MAC, we analyzed the expression of *C3* and *Hc*. A significant increase of *C3* mRNA expression was seen in six-week-old OSE mice (2.004-fold, p=0.006). We also found an upregulation of *C3* mRNA in eight-week-old OSE animals (2.465-fold, p=0.001; **Figure 7J**).

In accordance with the increase of *C3* mRNA, the expression of *Hc* mRNA encoding C5 was also upregulated at six weeks, in OSE mice (2.293-fold, p=0.004). At eight weeks, we noted a significantly higher *Hc* mRNA expression in the OSE group (1.711-fold, p=0.035; **Figure 7K**). Those results indicate an activation of the complement system in six- and eight-week-old OSE mice initiated by the classical pathway. Additionally, we noted an activation of the common pathway by the alternative pathway whereas the lectin pathway had no contribution to the activation of the complement system at any time.

### Correlation of Evaluated Parameters

Correlation analyses of SD-OCT parameters and RGC counts showed a correlation between ganglion cell complex thickness and RGC numbers at six weeks (r=0.5092; p=0.046; **Supplemental Figure 4A**). A stronger correlation for the same parameters was noted at eight weeks of age (r=0.5825; p=0.016; **Supplemental Figure 4B**). The total retinal thickness, evaluated via SD-OCT, also correlated with the RGC counts at six (r=-0.8211; p<0.001; **Supplemental Figure 4C**) and eight weeks of age (r=-0.8299; p<0.001; **Supplemental Figure 4D**). Regarding the OSE score at 56 days, a negative correlation was noted with RGC numbers at six weeks (r=-0.595; p=0.017; **Supplemental Figure 4E**), but not with RGC numbers at eight weeks (r=-0.3388; p=0.197; **Supplemental Figure 4F**). A strong negative correlation was observed between the clinical score at 56 days and the ganglion cell complex (evaluated *via* SD-OCT) at both time points, six (r=-0.8211; p<0.001; **Supplemental Figure 4G**) and eight weeks (r=-0.8299; p<0.001; **Supplemental Figure 4H**).

## Discussion

Understanding mechanisms and dynamics of degeneration of the visual system in chronic inflammatory conditions is a crucial step to implement effective therapeutic approaches, especially in rare conditions with potential devastating effects such as NMOSD or MOGAD. We therefore performed conclusive analyses of function, morphology, and histological alterations as well as changes of gene expression of inflammatory/immune pathways in a model of spontaneous encephalomyelitis. We provide evidence that early on during OSE, mice are affected by severe damage of the visual system with reduced function, retinal thinning, and severe inflammation with involvement of both the adaptive and innate immunity as well as activation of the complement system.

In autoimmune-mediated diseases, like MOGAD, NMOSD, or MS, neurodegeneration is a major component of the pathogenesis. Although the pathology of those diseases is not completely understood, neurodegeneration is mostly seen as secondary response to inflammatory-mediated demyelination (28, 29). Other studies refer to the role of neurodegeneration as a primary phenomenon occurring in myelin-deficient retina without optic neuritis (30, 31). In our study, OSE mice developed signs of encephalomyelitis after 26 days and 50% had a clinical score after four weeks. Intriguingly, we found indications of degeneration also in clinically unaltered animals without neurological signs.

The clinical analysis of the retina via OCT is frequently used as diagnostic tool in NMOSD and MOGAD (32). It is also applied to monitor disease progress in follow-up investigations (33). NMOSD patients have a significant reduction of the RNFL (34), correlating with the history of optic neuritis (35). In NMOSD, GCL and IPL are significantly more affected compared to MS or patients with isolated optic neuritis (36). Patterns of retinal thinning are, of note, similar in MOGAD and NMOSD (37). In contrast, the visual impairment due to retinal thinning is less severe in MOGAD compared to NMOSD (37–39).

Animal models offer a possibility to study dynamics longitudinally (40, 41). Cruz-Herranz et al. investigated the retinal changes in EAE mice, immunized with myelin oligodendrocyte glycoprotein peptide (MOG_35–55_). They found a thinning of the RNFL, the GCL, and IPL subsequent to the first signs of EAE (42). The reduction of those layers correlated with EAE severity and RGC loss. Another study also showed a significant reduction of the GCL in the EAE model eight weeks after MOG_35–55_ immunization (43). Until now, OCT has not been used in OSE. We showed that within a short time frame retinal thickness was further reduced from 8.2% in six-week-old OSE mice to 12.3% in eight-week-old animals. We especially noted a thinning of the ganglion cell complex via SD-OCT analysis, indicating that ganglion cells are predominantly affected. Also, we observed a strong correlation between retinal thickness and RGC counts. In addition, ganglion cell complex thickness and RGC cell numbers correlated. Our findings concur with the results from previous studies which describe a correlation of retinal thickness with the clinical score was well as with RGC counts (38, 43). This overall supports the use of OCT as marker of degeneration for progression and therapy response in OSE animal models as well as in patients.

Electroretinography is a well-established technique to investigate retinal function. Since retinal function can be impaired in NMO or MS patients with optic neuritis, ERG recordings were previously examined in patient studies (44, 45). ERG recordings were also analyzed in several mouse models for other diseases before (46, 47). We identified an impairment of retinal function correlating with retinal thinning in SD-OCT, suggesting an impairment of both the photoreceptors and the inner retinal layers.

Loss of RGCs correlates with irreversible neurodegeneration. We noted a significant loss of RGCs in the investigated OSE model, correlating with clinical progression. Zhang et al. noted a RGC decline in a rat NMOSD model, where animals were injected with AQP4 IgG-positive serum from NMOSD patients (48). Previous studies have described a significant RGC degeneration in several models of chronic EAE (19, 49–52). In a postmortem study of ocular pathology in MS patients, extensive neurodegeneration of the GCL and INL combined with inflammation of the retina was evident, irrespective of disease duration, while severity of retinal atrophy correlated with brain weight (53). Degeneration could be mediated by apoptosis (14, 19) or as result of inflammation during optic neuritis (54). We showed a reduction of RGCs both at six (37.8% reduction) and eight weeks (27.4% reduction), while OSE progressed further, suggesting that an initial peak of RGC degeneration is followed by rather moderate continuation of cell loss. This is highly relevant since we have shown previously in EAE that only a prophylactic treatment with the immunomodulatory agent laquinimod was able to reduce apoptosis and RGC loss, while a therapeutic approach failed (14).

The activation of macroglia, especially astrocytes, is a crucial factor in chronic inflammation in MS as well as in EAE (55, 56). In MS, an elevated GFAP level in the cerebrospinal fluid points towards severe astrogliosis (57). In contrast to MS, a decreased GFAP immunoreactivity was observed in NMO lesions, whereas the GFAP levels were increased in the cerebrospinal fluid due to the loss of astrocytes (58). The retina contains two types of macroglia, astrocytes and Müller cells (59). Surprisingly, we found no significant increase of the GFAP^+^ area in the retina at both time points. One possible explanation might be that astrocytic scar formation might happen later during the course of disease. This, however, remains to be proven with longitudinal investigations. Zeka et al. also observed little GFAP response in retinae of a rat model of NMOSD (11).

One hallmark of NMOSD and MOGAD is optic neuritis. The infiltration of the optic nerve with inflammatory cells has been shown in EAE (60) as well as in 12-week-old-mice OSE mice (9). In accordance with the results from these studies, we noted inflammatory infiltrations of the optic nerve, suggestive of optic neuritis, already in six- and eight-week-old-animals associated with demyelination. This was also accompanied by microglial activation, innate immune cells of the CNS important for homeostasis (61), phagocytosis, release of proinflammatory cytokines, and presentation of antigens to T cells (62). Microglia are crucially involved in the pathogenesis of chronic inflammation of the CNS, such as MS, especially during disease progression (63). Moreover, microglial interaction with astrocytes initiated by AQP4 antibodies leads to the formation of lesions in NMO (64). Although antibodies to AQP4 are absent in MOGAD, it is conceivable that microglia are also involved in the early formation of lesions in the OSE model.

In addition to the analyses of microglia in the optic nerve, we investigated microglia in the retina. Microglial activation of the retina was previously observed not only in NMO and MS, but also in EAE (64, 65). In OSE, active microglia and microglia/macrophages were significantly increased in the retina after six weeks. The analyses of the distribution of microglia/macrophages in the retinal layers showed that Iba1^+^ cells were only significantly increased in the GCL at six weeks whereas no differences were observed in the IPL and INL. In contrast, the number of Tmem119^+^ and Iba1^+^ microglia was increased in the GCL, IPL, and INL in the separate cell counts at six, but not at eight weeks suggesting an early peak in microglia response in OSE. In MS, CD68^+^ macrophages are upregulated in active white matter lesions in the brain (65). Interestingly, we found significantly upregulated expressions of CD68 and TNFα as well as TGFβ in the retina.

We also investigated the involvement of the complement system, since MOG IgG can induce complement dependent cytotoxicity (66). We showed, for the first time, an activation of the complement system, especially via the classical pathway, on mRNA and protein level. The alternative pathway was involved in the early phase of OSE whereas the lectin pathway was not involved, suggesting that the activation might be induced through antibody/antigen complex recognition. Further, microglia could be the source of complement, especially of C1q, in this model. In a mouse model for Alzheimer’s disease, a knock-out of C1q was accompanied by less microglia activation (67). The same group revealed that microglia are the dominant source of C1q in the brain and therefore probably also in the retina (68). The complement activation also supports the theory that neurodegeneration in the retina is not only a secondary phenomenon subsequent to isolated optic neuritis, but at least partly a result of the complement activation in the retina itself. Complement-dependent degeneration was described in the EAE model, while genetic lack of C3 protein protected from signs of EAE and neurodegeneration (69). Anti-complement directed therapeutic approaches such as the anti-C5 monoclonal antibody eculizumab are already in clinical use in NMOSD (70). While we showed an activation of the complement system in OSE, it remains so far unknown, whether activation of the complement system is a primary driver of pathology or rather an epiphenomenon of activated immune cells such as microglia. Hence, complement directed therapies should be investigated in animal models such as OSE to gather more data prior to potential use in MOGAD.

There are some limitations of this study that should be addressed. Only one control group, single-transgenic IgHMOG (Th) mice, was implemented in this study, based on the background of OSE mice. These control animals displayed some subclinical, histopathological alterations in optic nerves without developing neurological signs of EAE. Previous studies found no autoimmunity in the CNS of Th mice even in case of high MOG-autoantibody titers, which was explained by an intact blood-brain barrier (13), hence we suggested that autoimmunity will also be absent in the visual system. The noted subclinical histopathological alterations should be further investigated in future studies. A further limitation of this study is that mice were examined at certain ages and not disease progression stages. This should be addressed in further studies.

*In summary*, we provide evidence that OSE mice are affected by early damage of the visual system, including the retina and optic nerve, with altered retinal function, morphology, and evidence of inflammation, complement activation as well as degeneration. Since histological analyses of the retina or optic nerve in human are in general only feasible in postmortem tissue, those data help to understand dynamics of degeneration in MOGAD and NMOSD.

## Data Availability Statement

The original contributions presented in the study are included in the article/**Supplementary Material**. Further inquiries can be directed to the corresponding authors.

## Ethics Statement

The animal study was reviewed and approved by Landesamt für Natur, Umwelt und Verbraucherschutz Nordrhein-Westfalen (Recklinghausen, Germany; file no. 84-02.04.2016.A062).

## Author Contributions

LP, SR, SH, LD, and FG performed the experiments. LP, SR, SF, and SJ analyzed data. LP, SF, and SJ wrote the manuscript. SR, SH, FG, IK, HD, and RG critically revised the manuscript. SF and SJ designed and supervised the study. All authors read and approved the final manuscript.

## Funding

This study was supported by the Hertie Foundation and the FoRUM program of the Medical Faculty of Ruhr-University Bochum. We acknowledge the support by the Open Access Publication Funds of the Ruhr-University Bochum.

## Conflict of Interest

The authors declare that the research was conducted in the absence of any commercial or financial relationships that could be construed as a potential conflict of interest.

## Publisher’s Note

All claims expressed in this article are solely those of the authors and do not necessarily represent those of their affiliated organizations, or those of the publisher, the editors and the reviewers. Any product that may be evaluated in this article, or claim that may be made by its manufacturer, is not guaranteed or endorsed by the publisher.

## Abbreviations

AQP4, Aquaporin-4; CNS, Central nervous system; ERG, Electroretinography; GCL, Ganglion cell layer; GFAP, Glial fibrillary acidic protein; H&E, Hematoxylin and eosin; INL, Inner nuclear layer; IPL, Inner plexiform layer; LFB, Luxol fast blue; MAC, Membrane attack complex; MOGAD, Myelin oligodendrocyte glycoprotein-antibody-associated disease; MOG, Myelin oligodendrocyte glycoprotein; MS, Multiple Sclerosis; NMOSD, Neuromyelitis optica spectrum disorder; OCT, Optical coherence tomography; ONL, Outer nuclear layer; OSE, Opticospinal encephalomyelitis; RGC, Retinal ganglion cell; RNFL, Retinal nerve fiber layer; RT-qPCR, Quantitative real-time reverse transcription polymerase chain reaction.
