# Supplementary material for: Progressive Retinal and Optic Nerve Damage in a Mouse Model of Spontaneous Opticospinal Encephalomyelitis
Source: Front Immunol. 2022 Jan 24;12:759389. doi: 10.3389/fimmu.2021.759389 (PMC8818777; doi:10.3389/fimmu.2021.759389)
Supplement: Supplementary file 1 [file DataSheet_1.docx]

Supplementary Material

# Supplementary Figures


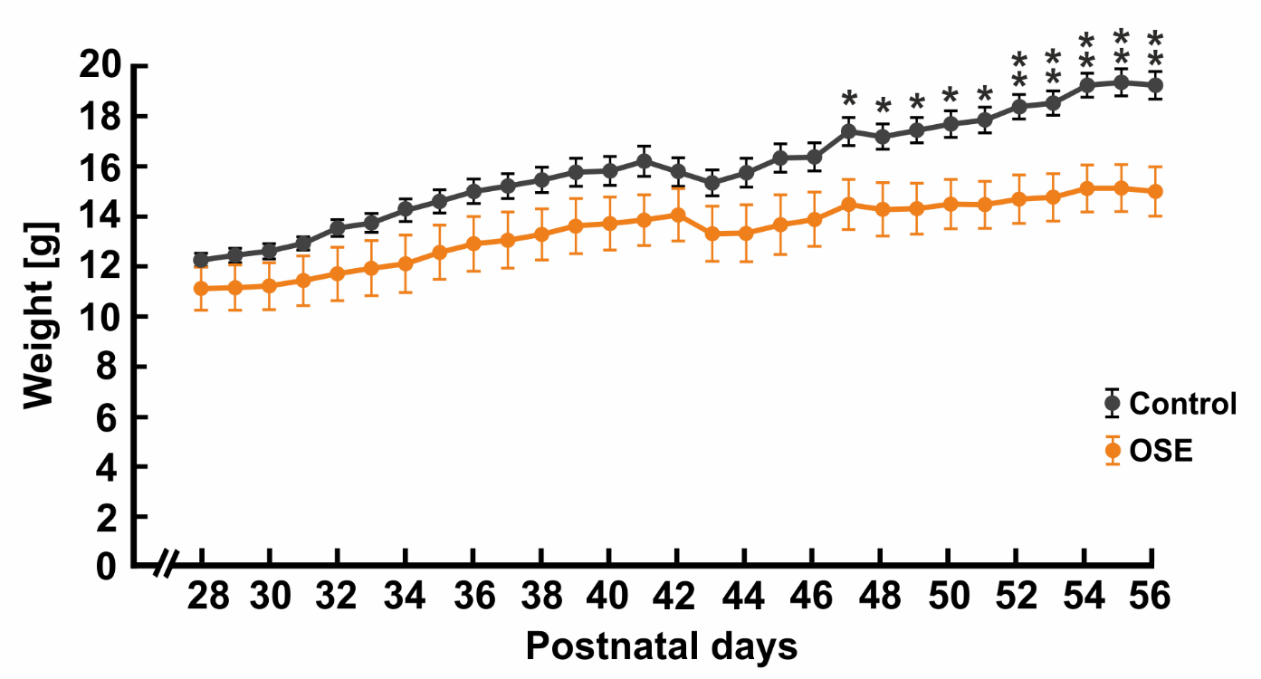


**Supplementary Figure 1: Lower weight of OSE mice.** Mice with clinical signs of opticospinal encephalomyelitis (OSE group) had a significantly reduced weight in comparison to the control group. Values are mean±SEM. *p<0.05, **p<0.01.

**
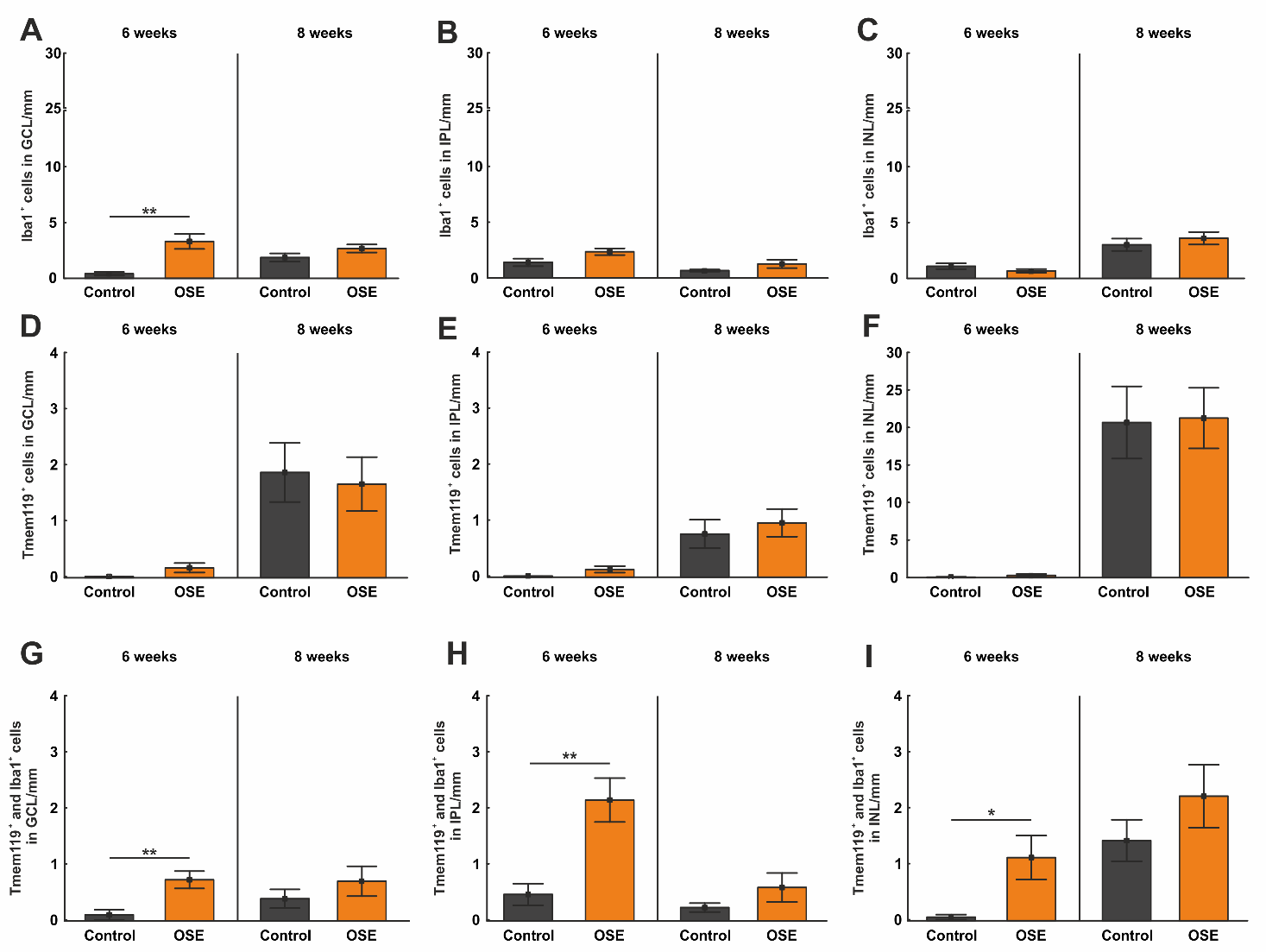
**

**Supplementary Figure 2: Higher microglia counts at 6 weeks. (A)** At 6 weeks, more Iba1^+^ cells were noted in the GCL of OSE mice compared to controls. **(B)** Iba1^+^ cells were comparable in the IPL and (**C.**) the INL. The number of Tmem119^+^ cells was similar in the GCL **(D)**, the IPL **(E)** and the INL **(F)** of both groups. **(G)** The number of Tmem119^+^ and Iba1^+^ cells was higher in the GCL in 6-week-old OSE mice in comparison to controls. **(H)** Also, the number of Tmem119^+^ and Iba1^+^ cells was higher in the IPL at 6 weeks. **(I)** This upregulation could also be observed in the INL at 6 weeks. Values are mean±SEM. Abbreviations: GCL=ganglion cell layer, IPL=inner plexiform layer, INL=inner nuclear layer. *p<0.05, **p<0.01.


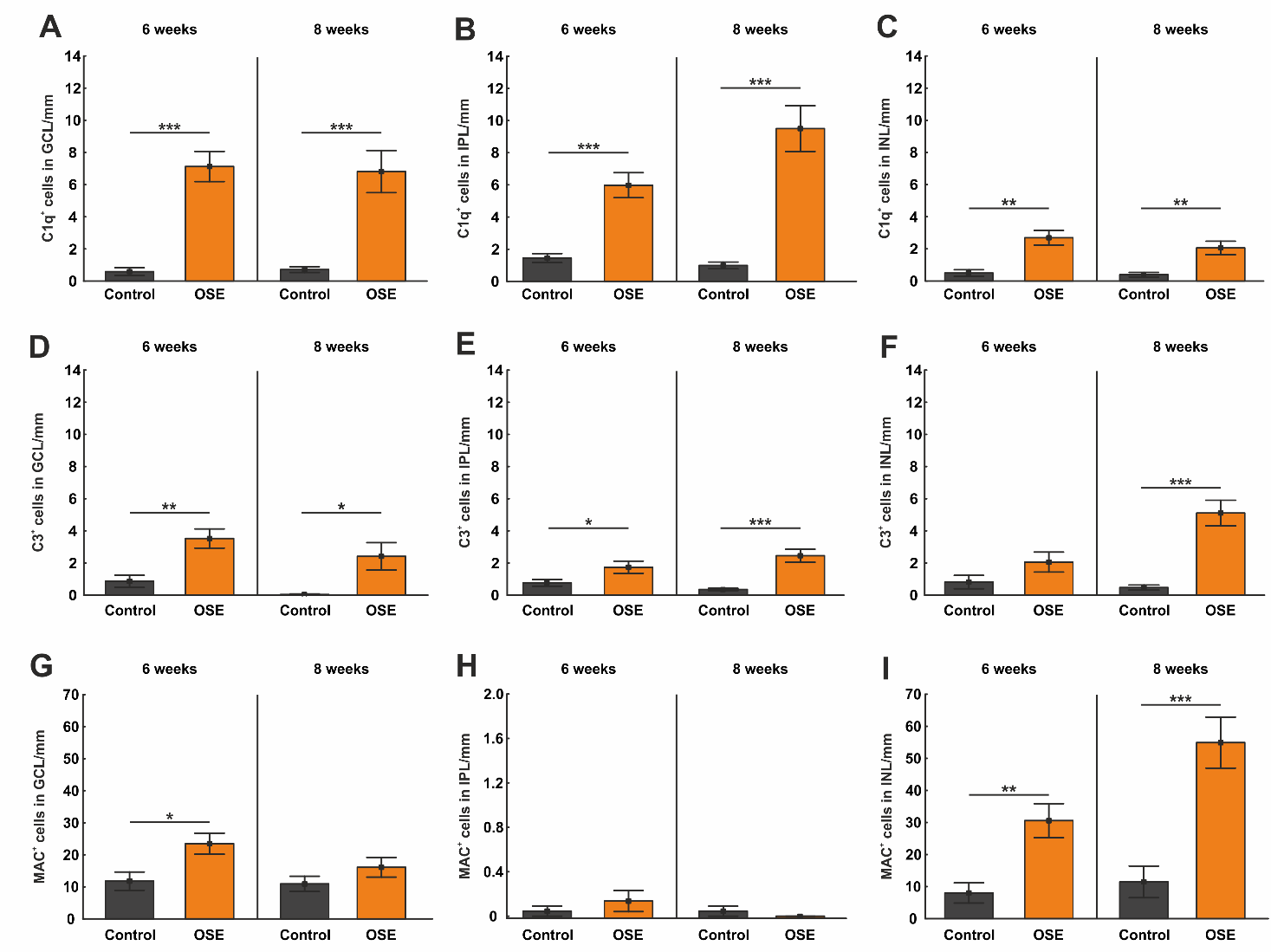


**Supplementary Figure 3: Increased complement factors in OSE retinae**. **(A)** More C1q^+^ cells were noted in the GCL of OSE mice at 6 and 8 weeks. **(B)** The IPL of the OSE group also displayed more C1q^+^ cells at both time points. **(C)** Significantly more C1q^+^ cells were seen in the INL. **(D)** OSE mice had more C3^+^ cells in the GCL at 6 and 8 weeks. **E.** In addition, more C3^+^ cells were observed in the IPL and **(F)** the INL. **(G)** The number of MAC^+^ cells in OSE mice was only significantly higher at 6 weeks. **(H)** Both groups displayed comparable numbers of MAC^+^ cells in the IPL. **(I)** More MAC^+^ cells were counted in the INL of OSE mice compared to controls at both time points. Values are mean±SEM. Abbreviations: GCL=ganglion cell layer, IPL=inner plexiform layer, INL=inner nuclear layer. *p<0.05, **p<0.01, ***p<0.001.


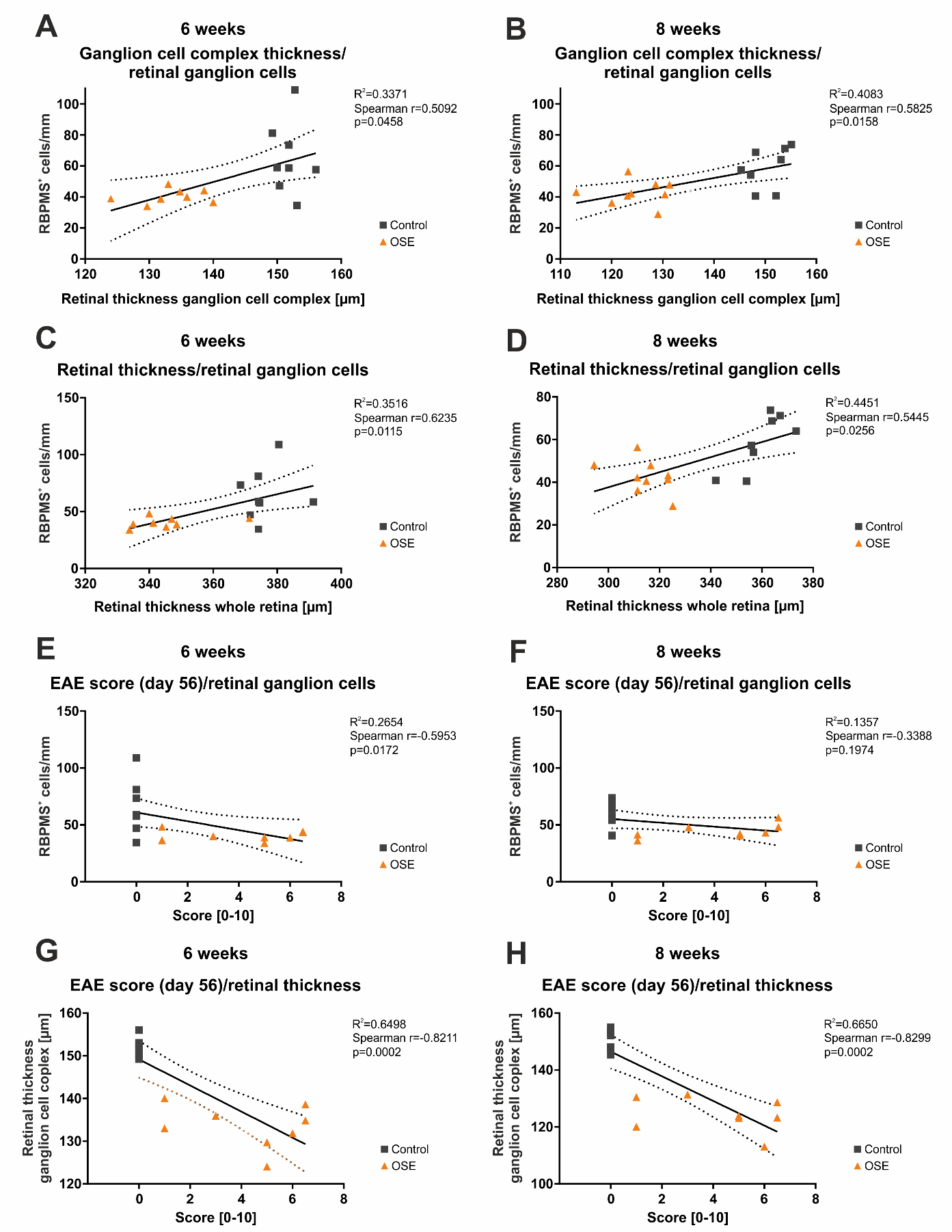


**Supplementary Figure 4: Correlation analyses. (A)** Correlations of SD-OCT parameters and retinal ganglion cell (RGC) counts showed a correlation between ganglion cell complex thickness and RGC numbers at 6 and **(B)** 8 weeks. **(C)** The total retinal thickness, evaluated via SD-OCT also correlated with the RGC counts at 6 and **(D)** 8 weeks. **(E)** Regarding the clinical score at 56 days, a correlation was noted with RGC numbers at 6 weeks. **(F)** This was not seen any more at 8 weeks. **(G)** A strong correlation could be noted between the clinical score at day 56 and the OCT analysis at 6 and **(H)** 8 weeks.
